# Drinking Over the Lifespan: Focus on College Ages

**DOI:** 10.35946/arcr.v38.1.13

**Published:** 2016

**Authors:** Jennifer E. Merrill, Kate B. Carey

**Affiliations:** Jennifer E. Merrill, Ph.D., is an assistant professor and Kate B. Carey, Ph.D., is a professor, both at the Center for Alcohol and Addiction Studies and the Department of Behavioral and Social Sciences at Brown University School of Public Health, Providence, Rhode Island

**Keywords:** Alcohol use, abuse, and dependence, alcohol use consequences, college drinking, heavy drinking, drinking patterns, college student, young adult, neurodevelopment, risk factors, protective factors, environmental factors, psychosocial factors, peer norms, parental support, college environment, prevention, intervention

## Abstract

Many college students drink heavily and experience myriad associated negative consequences. This review suggests that a developmental perspective can facilitate a better understanding of college drinking. Specifically, using an emerging adulthood framework that considers the ongoing role of parents and neurodevelopmental processes can provide insight into why students drink. Most college students drink and tend to drink more and more heavily than their non–college-attending peers. These drinking patterns are affected by environmental and temporal characteristics specific to the college environment, including residential campus living, the academic week, and the academic year. Additional psychosocial factors are of particular relevance to the drinking behavior of college-age people, and include exaggerated peer norms, the development and use of protective behavioral strategies, and mental health considerations. Understanding the unique interaction of person and environment is key to designing prevention/intervention efforts.

Approximately 41 percent of 18- to 24-year-olds are enrolled in a postsecondary degree-granting institution (National Center for Education Statistics 2013). As a group, college students, and particularly those at residential colleges (Presley et al. 2002), often drink heavily and experience myriad associated negative consequences. This selective review discusses the special characteristics of the college age and environment that put students at risk for hazardous drinking and problems with alcohol. The following sections describe the developmental context in which such drinking behavior occurs and then briefly characterize the risky drinking behavior of college students and the temporal and environmental risk factors associated with college attendance. The article then reviews psychosocial predictors of risky drinking that are relevant to this age group and concludes with intervention implications.

## Developmental Considerations

The developmental context in which drinking behavior occurs in college-aged men and women is unique, and developmental considerations can inform both basic and intervention research with this population.

### Emerging Adulthood

The sociodevelopmental notion of emerging adulthood is a helpful conceptual framework through which to understand risky drinking during the college years (Arnett 2000, 2005). For emerging adults who attend college, graduating from high school is no longer the entry into adulthood. Rather, these individuals typically delay marriage, parenthood, and a career until completing their education. Arnett describes five dimensions that characterize this developmental stage and that may have implications for alcohol use and misuse.

- Identity exploration. During emerging adulthood, when individuals are figuring out their own identity (particularly in the domains of love and work), alcohol use may be a part of exploring a wide range of lifestyle options before adopting adult roles and identity. Students may also use alcohol to cope with identity confusion (Schwartz et al. 2010).
- Instability. The college years are associated with frequent residential moves and changes in friends and partners, educational status, and jobs. Alcohol use often is elevated during periods of transition (Schulenberg and Maggs 2002) and perhaps is used for self-medication or to promote social activity (Kuntsche et al. 2005).
- Self-focus. Upon college entry, students gain independence from their family and relative freedom from obligations and commitments to others. They make independent decisions, and with weaker social controls from family and other institutions, they experience fewer constraints on risk behaviors. Friends may have the most influence on behavior during this time, and students inclined to use alcohol likely establish friendships that support drinking (Abar and Maggs 2010).
- Feeling in-between. Emerging adults may feel neither adolescent nor fully adult, and therefore may feel a sense of responsibility in some domains but not others. For example, they may feel capable of deciding whether or not to use alcohol but may not feel they need to conform to adult standards of comportment. Some students may see the college years as a “time out” from adult responsibilities (Colby et al. 2009) and give themselves permission to enjoy activities such as risky drinking that will be less acceptable later in adulthood.
- Possibilities. Finally, emerging adulthood is a time when people can make dramatic changes in their lives and is characterized by biased optimism. Because college students’ expectations for a positive future are so high, they may not acknowledge that negative consequences related to drinking behavior may occur.

### The Unique Role of Parents

As mentioned above, once emerging adults head to college, they depart from the structure and oversight provided when living with parents. However, parents do still matter during the college years. For example, research finds that higher levels of perceived parental involvement may buffer students from the effects of peers on alcohol use and problems (Wood et al. 2004); parental knowledge of how their college student is spending his or her time may influence choice of friends, which in turn may influence drinking behavior (Abar and Turrisi 2008); and parental permissiveness of drinking predicts increases in alcohol use and consequences over time (Walls et al. 2009). Overall, continued parental involvement and communication may serve to protect against high-risk drinking and prevent harm even at this stage of emerging adulthood (Turrisi and Ray 2010).

### Neurodevelopmental Factors Affecting Self-Regulation

The developmental context of college drinking is characterized not only by psychosocial but also biological factors. A growing body of research reveals that the brain’s frontal lobes do not fully mature until the mid-20s (Johnson et al. 2009). During adolescence, the bottom-up impulsive system that responds to rewards and social/emotional factors matures before the top-down controls of the prefrontal cortex (Casey and Jones 2010). Importantly, these top-down pathways from the prefrontal cortex help people slow down and consider the long-term outcomes of their behaviors. An imbalance between the impulsive system and the more reflective system may make emerging adults more vulnerable to engaging in addictive behaviors. In addition, some speculate that engaging in behaviors such as substance abuse may strengthen the bottom-up pathways and trigger this imbalance (Bechara 2005). Thus, the observations that late adolescents and emerging adults often choose short-term rewards over long-term goals may reflect the state of their neurocognitive development.

In the next section, we summarize descriptive data about college student drinking and its consequences, keeping in mind that it occurs within this developmental context characterized by the features of emerging adulthood, a changing but still significant role for parents, and continuing neurocognitive development.

## Alcohol Use and Consequences Among College Students

### Drinking Behavior

National surveys provide valuable data on the drinking habits of college students in the United States. They include the Harvard College Alcohol Study (e.g., Wechsler et al. 2002), the National Epidemiologic Survey on Alcohol and Related Conditions (e.g., Chen et al. 2004; Dawson et al. 2004), the National Survey on Drug Use and Health (Substance Abuse and Mental Health Services Administration 2014), the Core Institute Project (CORE), and the Monitoring the Future studies (Johnston et al. 2014).

White and Hingson (2013) offer a detailed overview of these surveys and their findings; we will provide a brief summary. To start, the majority of college students (approximately 60 percent) report past-month drinking (Johnston et al. 2014; Substance Abuse and Mental Health Services Administration 2014). Those who drink tend to drink heavily: more than one-third of college students report heavy episodic drinking at least once in the past 2 weeks, with heavy drinking defined as 4 or more drinks in one sitting for females and 5 or more drinks in one sitting for males (Johnston et al. 2014). In addition, approximately 1 of 5 males (19.9 percent) and 1 of 10 females (8.2 percent) consume twice this binge threshold (White et al. 2006). It is worth noting that patterns of drinking are heterogeneous with multiple trajectories in binge-drinking behavior across the 4 years of college (Schulenberg and Maggs 2002).

### Negative Consequences

Heavy drinking results in negative consequences for both drinking and nondrinking students:

- A total of 646,000 physical assaults, 97,000 sexual assaults, 599,000 unintentional injuries, and 1,825 deaths are linked to alcohol use among college students annually (Hingson et al. 2009).
- Forty percent of college student drinkers report alcohol-induced memory loss, such as blackouts (White et al. 2002), which is associated with future risk for injury and/or increased drinking (Mundt et al. 2012; Read et al. 2013).
- Twenty-one percent of college student drinkers report unplanned sexual activity while drinking, and 10 percent report unprotected sex while drinking (Wechsler et al. 2002). Such behavior can lead to sexually transmitted infections or unplanned pregnancy (Ingersoll et al. 2008).
- Students also report that drinking alcohol is related to social/interpersonal problems, poor self-care (e.g., eating and/or sleeping poorly), and diminished self-regard (e.g., feeling badly about oneself) (Read et al. 2006).
- Among college students, rates of alcohol abuse as defined in the *Diagnostic and Statistical Manual of Mental Disorders, Fourth Edition* range from 6 to 31 percent, and rates of alcohol dependence range from 6 to 16 percent (Blanco et al. 2008; Dawson et al. 2004; Knight et al. 2002).

From a developmental standpoint, the underdevelopment of the frontal lobes and neurocognitive systems guiding decision making may in part explain some of the consequences of drinking in this age group, particularly those that involve engaging in risky behaviors while drinking. Moreover, as mentioned, because expectations for a positive future are so high during emerging adulthood, college students may feel that they are immune to any negative consequences related to drinking and thus may not take measures to avoid them.

### Academic Impairment

Drinking also may influence students’ academics, the primary purpose of attending college. This may manifest in poor performance on exams, missing classes, lower grade-point average (GPA), and even dropping out (for a brief review, see White and Hingson 2013). However, the association between alcohol use and academics may be neither direct nor absolute. Although alcohol involvement has been shown to be associated with academic problems at the end of freshman year, this relationship was explained by historical variables (academic aptitude, class rank) that existed when students entered college (Wood et al. 1997). Binge drinking adversely affects GPA in part by reducing study hours (Wolaver 2002). Further, extreme alcohol involvement—dependence but not abuse—clearly compromises first-year academic performance (Aertgeerts and Buntinx 2002).

### Demographic Correlates

Just as in the general population, male and white students (Del Boca et al. 2004; Johnston et al. 2014) are at higher risk for excessive drinking. Certain affiliations associated with college life further enhance this risk, such as being a member of a Greek organization (O’Brien et al. 2013; Park et al. 2008) or a collegiate athletic team (Brenner and Swanik 2007; Yusko et al. 2008). Such affiliations are unique to the college environment and can be important sources of identity and social connectedness, which are both important to emerging adults.

### College versus Noncollege Comparisons

The drinking behavior among college students is in some ways distinct from that of their same-age peers who do not attend college (Slutske 2005; Slutske et al. 2004). Every year, from 2002 to 2013, rates of past-month binge drinking (4 or more drinks for women and 5 or more for men) were higher among college-attending young adults, ages 18–22, than their peers who do not attend college (Substance Abuse and Mental Health Services Administration 2014). Similar disparities are seen for alcohol use disorder, but some research finds that differences in alcohol use disorder disappear after adjusting for sociodemographic variables such as gender, race/ethnicity, nativity, marital status, and personal and family income (Blanco et al. 2008). Other studies find that variables such as full-time versus part-time status and type of college may be more directly related to variations in alcohol consumption than whether a student attends college (Carter et al. 2010). Selection factors associated with the type of college or choice of living situation may partly explain increased risk (Fromme et al. 2008). Nonetheless, college attendance provides an environmental context affording opportunities for high volume drinking. It also may prolong the sense of being in-between childhood and the responsibilities of adulthood.

## Risky Drinking Practices Among College Students

One explanation for increased risk for alcohol misuse and consequences among college students is the tendency to engage in specific types of high-risk drinking behaviors. These include but are not limited to pregaming and drinking games.

### Pregaming

Sometimes called “preloading,” “frontloading,” or “prepartying,” pregaming is defined as consuming alcohol before attending a social event, where additional alcohol may or may not be available and/or consumed (Read et al. 2010; Wells et al. 2009), and is common on U.S. college campuses. In fact, 70 to 75 percent of college drinkers report pregaming (Barnett et al. 2013; DeJong et al. 2010; Hummer et al. 2013; Pedersen and LaBrie 2007, 2008; Read et al. 2010) and say they engage in the practice on about one-third of drinking days (Labhart et al. 2013; Merrill et al. 2009; Read et al. 2010). Pregaming often takes place in college dorm rooms; is time limited because students need to leave for the primary event; and often involves doing shots of hard liquor, resulting in rapid rates of intoxication (DeJong et al. 2010). When students pregame, compared with drinking episodes when they do not, they consume a greater number of drinks and have higher blood alcohol concentrations (BACs) (Barnett et al. 2013; Borsari et al. 2007*a*; Glindemann et al. 2006; LaBrie and Pedersen 2008; Pedersen and LaBrie 2007; Read et al. 2010). In addition, pregaming is linked to more alcohol-related consequences (Kenney et al. 2010; Labhart et al. 2013; LaBrie and Pedersen 2008; Merrill et al. 2013*a*; Paves et al. 2012; Pedersen et al. 2009), including neglecting responsibilities, feeling sick, passing out, absenteeism at school/work, drunk driving, alcohol poisoning, aggressive or violent acts, and blackouts (DeJong et al. 2010; Hughes et al. 2008; LaBrie and Pedersen 2008; LaBrie et al. 2011; Pedersen and LaBrie 2007; Pedersen et al. 2009).

### Drinking Games

Another common and risky practice is playing drinking games (such as beer pong and Kings). According to Zamboanga and colleagues (2013), drinking games involve performing some kind of cognitive and/or physical task, are governed by a set of rules that specify when and how much participants should drink, and are designed specifically to promote increased drinking within short time periods in a social setting. In some cases, a vicious cycle can occur wherein once a participant starts to lose, he or she is forced to drink more as a penalty, thus further diminishing his or her skills in the game and increasing required consumption (Zamboanga 2007*a*; Zamboanga et al. 2010). Individuals may be heckled for refusing to drink during the game (Borsari 2004). It is therefore not surprising that playing drinking games increases risk for heavy drinking and negative alcohol-related outcomes (Ray et al. 2014; Zamboanga et al. 2006). One category of drinking games, including chugging and keg stands, is referred to as consumption or extreme consumption games (Zamboanga et al. 2013). It is this category that may pose the greatest risk for elevated alcohol consumption (LaBrie et al. 2013; Zamboanga et al. 2006, 2007*b*).

## Environmental and Temporal Risk Factors for College Students

College attendance places students at increased risk for alcohol consumption and alcohol-related problems in part because of environmental features, including communal living and an academic week that often allows students to select a schedule with long weekends. Furthermore, the rhythm of the academic year includes social holidays and events that happen predictably across college campuses.

### Living Situation

Communal living is an important risk factor. For example, Zamboanga and colleagues (2009) found that students at a women’s liberal arts college who lived in residence halls reported higher levels of hazardous alcohol use than students living in house-style residences, and Willoughby and Carroll (2009) demonstrated that students living in co-ed housing were more likely than students living in gender-specific housing to binge drink and consume alcohol. In contrast, students who remain living at home with parents drink less (Valliant and Scanlan 1996). In other work, alcohol dependence rates were highest among college students of both genders who live on campus, and rates of alcohol abuse were highest among college men who live off campus (Dawson et al. 2004, 2005*a*). Within the context of the emerging adulthood framework, living situation during the college years can contribute both to instability (frequent moves) and self-focus (weaker social controls upon moving from home to dormitories where the influence of parents may decline and influence of friends may rise).

### The Transition Into College

The transition from high school to the first year of college is associated with increases in alcohol use and heavy drinking (Borsari et al. 2007*b*; Sher and Rutledge 2007). Heavy or frequent drinking early in the college experience can compromise academic success (Hoeppner et al. 2012; Upcraft 1995), as problematic patterns of drinking established during the first weeks often continue throughout college (Schulenberg et al. 2001; Task Force of the National Advisory Council on Alcohol Abuse and Alcoholism 2002). A review by Borsari and colleagues (2007*b*) found that the risk for increased drinking associated with college attendance is moderated by a number of variables, including sensation seeking, race, gender, religiosity, precollege alcohol use, and parental influences. This risk also is explained in part by changes in determinants of drinking that occur upon college entry, including changes in alcohol expectancies, drinking motives, perceived norms, Greek membership, and drinking game participation. The emerging adulthood framework predicts increased drinking during this time frame in that alcohol use is used to cope with the need to rebuild a social life or recreate a social identity; alcohol use also can be a result of an enhanced susceptibility to peer influence (Arnett 2005).

### The Academic Week

College students typically drink the heaviest on weekends, and, for some, weekend-like drinking begins on Thursday (Hoeppner et al. 2012). However, this trend is moderated by a student’s schedule; those with no Friday classes drink twice as much on Thursdays as students with early Friday classes (Ward et al. 2013; Wood et al. 2007). Most colleges afford students the ability to select their own schedule, so heavy drinking students may be least likely to enroll in classes that convene on Friday (Paschall et al. 2006), perhaps in an effort to seek more opportunities to drink.

### The Academic Year

Importantly, patterns of drinking across the academic year are not uniform; multiple trajectories characterize the overall pattern of drinking across the first college year (Greenbaum et al. 2005). However, at least among first-year students, some of the heaviest drinking occurs not only during the initial weeks of fall semester, as described above, but also during the initial weeks of spring semester (Del Boca et al. 2004; Tremblay et al. 2010). In contrast, the lightest drinking occurs during exam weeks, both midterms and finals (Del Boca et al. 2004). As described below, research also reveals that the heaviest drinking takes place on holidays and during holiday breaks when students are not on campus.

### Holidays and Breaks

Drinking among freshmen peaks during Spring Break, Thanksgiving, Christmas, and New Year’s weeks (Del Boca et al. 2004) and tends to be characterized by binge drinking (Beets et al. 2009; Greenbaum et al. 2005). A study of 21-year-old college students found that compared with a typical nonholiday weekend, more students consumed alcohol and they reached higher BACs on New Year’s Eve, New Year’s Day, July 4th, Spring Break, and graduation (Neighbors et al. 2011).

Drinking and related consequences are higher during Spring Break than the typical week (Beets et al. 2009; Del Boca et al. 2004); however, this is particularly true for students who go on trips with friends (Grekin et al. 2007; Lee et al. 2006, 2009). The highest levels of drinking during Spring Break occur among those who report higher levels of intentions to drink before their trip, those who go on longer trips, and those who previously engaged in more heavy episodic drinking (Patrick and Lee 2012). Notably, however, students who typically drink less experience more negative consequences of drinking during Spring Break (Lee et al. 2009).

### 21st Birthdays

Extreme drinking and negative alcohol-related consequences also are associated with 21st birthdays (Lewis et al. 2009; Neighbors et al. 2005; Rutledge et al. 2008), which typically occur during students’ college years. Half of 21st-birthday drinkers consume more on this day than any other prior occasion (Rutledge et al. 2008), and students drink more than they anticipate they will at this celebration (Brister et al. 2010). Students who do not typically drink heavily but do so the week of their birthday are most likely to experience higher levels of alcohol-related consequences (Lewis et al. 2009). In addition, 21st-birthday drinking is associated with the highest proportion of drinkers and highest BACs compared with other high-risk times (Neighbors et al. 2011).

### Campus Events

Drinking also tends to spike during campus- or university-specific events. For example, during “State Patty’s Day,” a student-constructed, party-focused holiday at Pennsylvania State University, first-year students were more likely to drink and drink heavily (Lefkowitz et al. 2012). On this day, students consumed more alcohol than on other weekend days, even after controlling for gender and drinking motives, and local crime rates increased.

Sporting events also are associated with heavy drinking among college students (Glassman et al. 2010; Neal and Fromme 2007) and also seem to increase risk for consequences. For example, college football homegame days see a 9 percent increase in assaults, a 41 percent increase in arrests for alcohol-related disorderly conduct, and a 76 percent increase in liquor-law violations compared with nongame days (Rees and Schnepel 2009). High-profile sporting events (e.g., winning an NCAA championship) increase game-day drinking on average and more so for heavier and more impulsive drinkers (Neal et al. 2005).

### The Transition Out of College and Into Adulthood

Despite the often risky nature of drinking during college, most, although not all, students “mature out” of such behavior (Littlefield et al. 2009). The average decline in drinking behavior following the college years has been attributed to events that are delayed for emerging adults who choose to attend college, namely employment, marriage, and parenthood, each of which may accompany reductions in recreational and social activities that involve drinking (Gotham et al. 2003; O’Malley 2004). Age-related changes in personality also may be associated with reductions in drinking during adulthood (Littlefield et al. 2009). However, students with alcohol use disorder are at higher risk for maintaining problematic drinking patterns: about one-half of students who meet alcohol use disorder criteria at age 19 maintain that status at age 25 (Rohde et al. 2001; Sher and Gotham 1999).

## Psychosocial Determinants of Drinking During the College Years

College student drinking is affected by several psychosocial determinants that also influence drinking behavior in similar ways during other developmental periods. For example, like the general populations, college students tend to drink more if they believe drinking will have positive effects and consequences, and they tend to drink less if they have negative expectations about drinking (e.g., Gaher and Simons 2007; Wardell and Read 2013). In addition, how positively or negatively students view the expected effects of alcohol (Gaher and Simons 2007) or view actual recently experienced consequences of drinking (Merrill et al. 2013*b*) are also important predictors of college drinking.

A person’s reasons or motives for drinking also influence their alcohol use. For example, drinking to increase positive affect, called enhancement motives, consistently predicts alcohol use and tends to be linked to negative alcohol consequences indirectly, through higher drinking levels (Magid et al. 2007; Merrill and Read 2010; Read et al. 2003). Meanwhile, drinking to alleviate negative affect, or coping motives, are directly associated with negative alcohol consequences in college students (Jones et al. 2014; Kassel et al. 2000; Merrill and Read 2010; Merrill et al. 2014). Certain personality characteristics, such as sensation seeking or impulsivity (Diulio et al. 2014; Kazemi et al. 2014*a*) and neuroticism (Martin and Sher 1994; Vollrath and Torgersen 2002), have been linked to increased drinking behavior among college students, although findings are mixed. In addition, a person’s drinking level prior to entering college predicts drinking behavior during college (Sher and Rutledge 2007; Varvil-Weld et al. 2013).

Below we discuss in more detail common psychosocial determinants that exert influence in a way that is unique to the college years. We highlight exaggerated norms, protective behavioral strategies, and mental health.

### Exaggerated Norms

Peers influence young adult drinkers in several direct and indirect ways (Borsari and Carey 2001). Perhaps the most studied has been young adults’ perceptions of drinking norms. In fact, when comparing their own drinking behavior (their personal norms) with their perceptions of how much or how often other students drink (descriptive norms) and their perceptions of whether peers approve of drinking and related behaviors (injunctive norms), young adults tend to see others as drinking more and more approving of drinking (Borsari and Carey 2003). When objective evidence of peer drinking is available, the perceived drinking norm is invariably overestimated (e.g., Carey et al. 2006). Research demonstrates the importance of reference group: norms for close friends are more highly correlated with student drinking behavior than those of more distal student groups (Larimer et al. 2009; Neighbors et al. 2008). However, providing students with corrective feedback on drinking norms for other relevant peer groups, because they often are objectively exaggerated, can promote discrepancies that lead to drinking reductions (Larimer et al. 2009). Descriptive and injunctive norms seem to have unique influences on drinking behavior (Larimer et al. 2004). In fact, descriptive norms have a greater influence when there are also permissive injunctive norms, positive outcome expectancies, and higher identification with the referent group (Neighbors et al. 2010; Rimal 2008). The peer-intensive nature of college life affords many opportunities to affiliate with groups that develop their own normative cultures related to drinking (e.g., Greeks, athletic teams, and clubs). Within the context of the emerging adulthood framework, norms are relevant to the factors of both identity exploration (looking to others in the social environment while figuring out his or her own identity) and self-focus (friends as most influential on behavior during this age).

### Protective Behavioral Strategies

In light of all of the contextual and developmental factors that contribute to risk described above, it is essential that students learn to drink safely (if they choose to drink) when navigating the novel drinking environment of college. However, the extent to which college students acquire and use safe drinking skills varies. Most emerging adults leave home for college before they attain the minimum legal drinking age. Thus, peers and not parents or other adults often serve as the primary sources for learning how to drink. Protective behavioral strategies—techniques that can be used to minimize harm associated with alcohol use such as setting drink limits, consuming nonalcoholic in addition to alcoholic drinks, avoiding drinking games, and using a designated driver—have received an increasing amount of attention in the college-drinking literature over the past few decades. A recent review highlights several studies that consistently reveal that individuals who report using more protective behavioral strategies also report drinking less and/or experiencing fewer alcohol-related problems (Pearson 2013).

### Mental Health

Approximately three-quarters of lifetime mood or anxiety disorders begin by age 24, coinciding with the typical college years (Kessler et al. 2005), and about 11 percent and 12 percent of U.S. college students meet criteria for mood and anxiety disorders, respectively (Blanco et al. 2008). Unfortunately, few college students use mental health services (e.g., Eisenberg et al. 2011), and research finds an association between mental health problems and heavy episodic drinking (Cranford et al. 2009). Moreover, students with mental health symptoms are more likely to experience problems related to alcohol use than students without such symptoms, regardless of drinking level (Dawson et al. 2005*b*; Dennhardt and Murphy 2011; Kenney and LaBrie 2013; LaBrie et al. 2010; Weitzman 2004). Within the emerging adulthood framework, mental health issues are relevant to the factors of both identity exploration (identity confusion may cause distress) and instability (transitions may be disruptive), as alcohol may be used for self-medication purposes among students high on either dimension.

## Intervention Implications

The findings reviewed above have several implications for interventions with the special population of college-aged individuals. In general, a harm prevention/harm reduction approach, as opposed to an abstinence-based approach is considered most appropriate for young people who are developing drinking habits and have not exhibited signs of dependence (Ehret et al. 2013; Marlatt and Witkiewitz 2002). Also, given that aspects of the campus environment constitute risk factors for individual drinkers, it is important to implement not only coordinated alcohol abuse prevention efforts involving community and campus environmental management but also group and individual prevention efforts and to identify drinkers in need of treatment services (Toomey et al. 2013; Wolfson et al. 2012). The next section reviews how prevention and intervention efforts can incorporate the patterns and influences we describe above.

### Developmental Factors

Despite the importance of the developmental context to college student drinking, to date, developmental considerations have had limited influence on intervention development. A notable exception is parent-based intervention, which has been well received and shows promise both as a standalone intervention (Ichiyama et al. 2009; Turrisi et al. 2013) and a supplement to student-based interventions (Turrisi et al. 2009). In line with the emerging adulthood concept of “possibilities,” interventions highlighting future academic and occupational decisions also may be useful. An example of this comes from a study that modified a traditional brief motivational intervention to include a supplemental session focused on increasing the salience of academic and career goals and discussed behavior patterns that would assist in meeting those goals (Murphy et al. 2012). Students who received the supplement reported fewer alcohol-related consequences at 1- and 6-month followups compared with students who did not receive the supplement. In addition, interventions can address self-regulatory difficulties associated with incomplete prefrontal control by using mobile technologies, which permit real-time assessment (e.g., Mays et al. 2010) and interventions delivered close to drinking events (e.g., Suffoletto et al. 2012). Such approaches seem to be both feasible and acceptable to college students (Kazemi et al. 2014*b*). Additional adaptation of the content and delivery of interventions based on the developmental context of college drinkers is a promising direction for intervention development.

### Environmental and Temporal Factors

Tailoring interventions to address environmental issues of the college setting also may be beneficial. Such interventions include establishing substance-free residential options and changing the academic schedule to ensure that students take classes on Fridays and also in the mornings (DeJong and Langford 2002). Increased regulation and/or detection of alcohol use among underage drinkers in particular may be needed at campus events such as football games. Toomey and colleagues (2007) provide a more detailed review of these and other strategies designed for environmental management.

Event-specific prevention (ESP) is an intervention strategy that addresses temporal determinants of drinking behavior (Neighbors et al. 2007). ESP assumes that knowing when and/or where risky drinking will occur provides an opportunity for its prevention. For example, knowing that 21st birthdays and Spring Break are times of greatest risk suggests that resources should be allocated toward prevention around these times, providing a cost-effective approach to preventing alcohol-related consequences associated with these events (Neighbors et al. 2011, 2012). Finally, early preventive interventions for first-year students transitioning into college may help thwart increases in risky drinking behavior. There is modest support that online educational programs are effective for these students (Hustad et al. 2010; Lovecchio et al. 2010). Further, meta-analytic research suggests that behavioral interventions for first-year college students effectively reduce alcohol consumption and alcohol-related problems, with the extent of reductions dependent on intervention content (e.g., personalized feedback provides better outcomes) (Scott-Sheldon et al. 2014).

### Psychosocial Determinants

Many of the psychosocial determinants of drinking during emerging adulthood reviewed above have informed the development of alcohol abuse prevention interventions. For example, correcting exaggerated perceived norms is a well-documented active ingredient of successful risk-reduction programs delivered both in person and by computer (Carey et al. 2010; Doumas et al. 2009; Neighbors et al. 2004; Turrisi et al. 2009). Further, interventions increasingly are incorporating protective behavioral strategies (Pearson 2013), which have been shown to mediate intervention effects (Barnett et al. 2007; Larimer et al. 2007; Murphy et al. 2012).

### Future Directions for Intervention

Tailoring alcohol risk reduction interventions to students with mental health concerns would be another way to integrate psychosocial determinants of drinking and the emerging adulthood framework into new interventions. For example, interventions that provide alternatives to substance use for coping with negative mood states could prove fruitful for students high on the instability dimension of emerging adulthood and who are experiencing negative affect related to transitions, or for students who experience identity confusion during this time of exploration. In addition, recent data demonstrate that depression may interfere with intervention-related change (Geisner et al. 2015; Merrill et al. 2014). Although the exact mechanisms of this effect are as yet unknown, it may be beneficial to include in brief interventions components that seek to increase substance-free reinforcement (e.g., Murphy et al. 2012) or that broaden students’ coping skills.

To date, we have no evidence-based interventions to reduce high-risk practices such as pregaming or drinking games (Read 2014). Such interventions might involve education about factors affecting BAC and the biphasic curve to help sensitize some drinkers to the risk of consuming large quantities in a short time; corrective normative feedback about the frequency, intensity, or approval of high-risk behaviors by peers; and/or the provision of protective behavioral strategies specific to refusing opportunities to pregame or learning to play drinking games safely.

## Conclusion

Much progress has been made in understanding the risk for alcohol misuse among college students. However, there still is room to understand the developmental, social, and environmental factors influencing college student drinking, to best design interventions that can ultimately reduce harm for this special population.
